# Medical dispatchers recognise substantial amount of acute stroke during emergency calls

**DOI:** 10.1186/s13049-016-0277-5

**Published:** 2016-07-07

**Authors:** Søren Viereck, Thea Palsgaard Møller, Helle Klingenberg Iversen, Hanne Christensen, Freddy Lippert

**Affiliations:** Emergency Medical Services Copenhagen, University of Copenhagen, Telegrafvej 5, 2750 Ballerup, Denmark; Stroke Unit, Department of Neurology, Copenhagen University Hospital, Rigshospitalet-Glostrup, Nordre Ringvej 57, 2600 Glostrup, Denmark; Stroke Unit, Department of Neurology, Copenhagen University Hospital, Bispebjerg, Bispebjerg Bakke 23, 2400 Copenhagen, NV Denmark

**Keywords:** Stroke, Emergency Medical Services, Medical decision making, Emergency Medical Dispatch

## Abstract

**Background:**

Immediate recognition of stroke symptoms is crucial to ensure timely access to revascularisation therapy. Medical dispatchers ensure fast admission to stroke facilities by prioritising the appropriate medical response. Data on medical dispatchers’ ability to recognise symptoms of acute stroke are therefore critical in organising emergency stroke care.

We aimed to describe the sensitivity and positive predictive value of medical dispatchers’ ability to recognise acute stroke during emergency calls, and to identify factors associated with recognition.

**Methods:**

This was an observational study of 2653 consecutive unselected patients with a final diagnosis of stroke or transient ischemic attack (TIA). All admitted through the Emergency Medical Services Copenhagen, during a 2-year study period (2012–2014). Final diagnoses were matched with dispatch codes from the Emergency Medical Dispatch Centre. Sensitivity and positive predictive value were calculated. The effect of age, gender, and time-of-day was analysed using multivariable logistic regression.

**Results:**

The sensitivity was 66.2 % (95 % CI: 64.4 %–68.0 %), and the positive predictive value was 30.2 % (95 % CI: 29.1 %–31.4 %). The multivariable logistic regression analyses showed that emergency calls during daytime and a final diagnosis of TIA vs. intracerebral haemorrhage (ICH), was positively associated with recognition of stroke (OR 2.70, 95 % CI: 2.04–3.57).

**Discussion:**

This study reports a high rate of stroke recognition compared to other studies ranging from 31% to 74%. The high sensitivity is likely the result of a profound reorganisation of the Emergency Medical ServicesCopenhagen, including the introduction of EMDs with a medical profession, and a criteria-based dispatch tool. A recognition rate of 100 % is not obtainable without an inappropriate amount of false positive cases.

**Conclusions:**

We report an overall high recognition of stroke by medical dispatchers. A final diagnosis of TIA, compared to ICH, was positively associated with recognition of acute stroke. Emergency medical dispatchers serve as the essential first step in ensuring fast-track stroke treatment, which would promote timely acute therapy.

**Trial registration:**

Unique identifier: NCT02191514.

**Electronic supplementary material:**

The online version of this article (doi:10.1186/s13049-016-0277-5) contains supplementary material, which is available to authorized users.

## Background

Intravenous thrombolysis treatment reduces long-term mortality [1] and is proven efficient and safe up to 4.5 h after onset of acute ischemic stroke (AIS) [2]. Moreover, patients’ morbidity and mortality after AIS is dependent on the time from symptom-onset to definitive care [3–5]. In-hospital time reduction for stroke patients has been an area of great progress. The median time from arrival at the hospital to thrombolysis treatment is reported as short as 21 min [6].

Despite a safe and effective treatment, the amount of AIS patients receiving thrombolysis ranges between 11 % and 18 % in Denmark [7]. Excess time from symptom-onset to in-hospital evaluation is reported the main reason why physicians refrain from thrombolysis [8].

Time from symptom onset to interventional treatment depends on patient awareness of emergency symptoms and contact to the emergency medical services (EMS) through an emergency call. Transportation by EMS, as opposed to private transport, and pre-arrival notification of the hospital stroke team is associated with shorter prehospital and in-hospital time respectively [9, 10]. Therefore, provision of the adequate ambulance response dispatched from the Emergency Medical Dispatch Centre (EMDC) is essential.

A vital point in this work flow is recognition of stroke by the Emergency Medical Dispatcher (EMD) [11]. Recognition of stroke is a prerequisite for the necessary prehospital assessment, prehospital notification of the in-hospital stroke team, and appropriate transport to the hospital — ultimately ensuring the best patient care as recommended in Danish and international guidelines [12, 13]. In order to improve stroke recognition, it is vital to report results from EMDCs around the world and to explore barriers for recognition of stroke. This has the potential to increase the EMDs awareness when handling stroke patients, as well as the future development of dispatch systems.

The aim of this study was two-fold:

1. To determine the sensitivity and the positive predictive value of stroke recognition by EMDs during emergency calls
2. To evaluate if patient’s sex, time of day, or final diagnosis was associated with stroke recognition

## Methods

### Study design

We performed an observational register-based study. To determine if the EMD recognised the stroke during the emergency call, we studied dispatch codes from the EMDC. We included patients registered at hospital discharge with a final main diagnosis of acute ischemic stroke (AIS), intracerebral haemorrhage (ICH), or transient ischemic attack (TIA).

This study included TIA, ICH, and AIS but excluded subarachnoid haemorrhage (SAH). The main focus of this study was recognition of symptomatology, in which SAH differs substantially. The term “stroke” will refer to ICH and AIS throughout the paper.

### Setting

The study was conducted in the Capital Region of Denmark, covering the Copenhagen area and the surrounding suburbs. It inhabits 1.75 million people in an area of 2561 km^2^.

Thrombolysis treatment is centralised in two stroke centres, which are responsible for the stroke fast track care on alternate days.

### EMS setting

In Denmark, one emergency phone-number (1-1-2) leads to a common call centre that handles police, fire, and medical emergencies. In case of a medical emergency, the caller is forwarded to an EMDC where an EMD handles and prioritises the call. To assist the EMD, an electronic criteria-based decision tool (Danish Index for Emergency Care) [14] is available. All EMDs are either registered nurses or paramedics. They have received 6 weeks of additional training in communication and the use of Danish Index for Emergency Care. The tool suggests a default priority of response ranging from A to F, depending on the caller’s main complaint and urgency of the condition. Priority A is the most acute response and priority F is non-urgent. The EMDC Copenhagen handles approximately 105,000 emergency calls annually. In case of suspected stroke, the default response is a priority “A” ambulance manned with either paramedics or emergency medical technicians. Neither the EMDs or the ambulance personnel uses a strict standardised tool, like the Los Angeles Prehospital Stroke Screen [15], for evaluation of stroke symptoms.

### Prehospital response setting

When the ambulance personnel suspects a stroke within the time window for thrombolysis, they consult the stroke neurologist on call at the stroke centre. If the patient is considered eligible for thrombolysis, the patient will be transported to the stroke centre for further examination.

The median response time for patients suitable for thrombolysis (priority A) in Copenhagen is five minutes [16].

### Participants

**Fig. 1:**
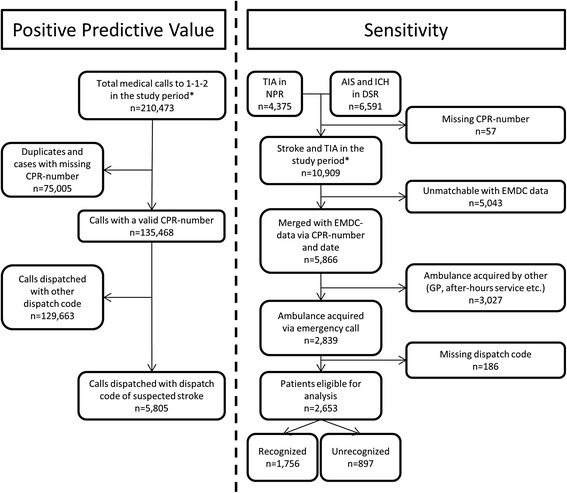
Flowchart describing the participants divided in the calculation of sensitivity and positive predictive value. *Study Period: 1^st^ of January 2012–31^st^ of December 2013. NPR: National Patient Registry, DSR: Danish Stroke Registry, CPR-number: Unique Personal Identification number, TIA: Transient Ischemic Attack, AIS: Acute Ischemic stroke, ICH: Intracerebral Haemorrhage, GP: General Practitioner, EMDC: Emergency Medical Dispatch Centre

We used two data sources for identification of stroke patients: The National Patient Registry (NPR) [17] and the Danish Stroke Registry (DSR) [18]. Patients registered with International Classification of Diseases, Tenth Revision (ICD-10) codes, corresponding to TIA (DG459), ICH (DI61) or AIS (DI63) in the period from 1^st^ of January 2012 to 31^st^ of December 2013, in the Capital Region of Denmark were included. At the time of study, TIA was not registered in the DSR, therefore data on TIA was collected from the NPR (see Fig. 1). To evaluate if stroke was recognised by the EMD, we identified the patients in the EMDC database using the unique Danish civil registration number (CPR-number) [19] and date of admission as a unique key for data merging. Exclusion criteria were: If CPR-number was missing, if patients were registered in the DSR with a diagnosis of “unspecified stroke (DI64) “, if the case could not be merged with EMDC data, or if patients were transported to the hospital by an ambulance not requested through an emergency call.

### Derived variables

- The following ICD-10 codes were identified in the NPR: DG459 (TIA), and DSR: DI61 (ICH) and DI63 (AIS).
- Sex and age were derived from the CPR-number, which was registered during the emergency call and upon arrival at the hospital. Age was registered as a continuous variable.
- For calculation of diurnal variation, three time - intervals were defined: Night (23:00–06:59), day (07:00–14:59), and evening (15:00–22:59).
- Dispatch codes and response priority were obtained from the EMDC database. A dispatch code is assigned for each emergency call, containing information about urgency of the condition and main complaint. The following dispatch codes from the Danish Index for Emergency Care were considered as suspected stroke: A.26.03:” Lowered consciousness-paralysis, suspected stroke: Suddenly asymmetry in the face, lowered force in arms/legs, and/or verbal difficulties” or A.26.04: “Lowered consciousness-paralysis, increasingly unclear/ listless-suspected stroke”. All other dispatch codes were defined as “not stroke”.

### Statistical methods

To investigate recognition of TIA, ICH, and AIS by EMDs, sensitivity and positive predictive value (PPV) with 95 % confidence intervals (CI) were calculated. To analyse factors associated with recognition of stroke; sensitivity of stroke recognition within final diagnoses, diurnal variations, and sex were compared with Chi2-test, and multivariable logistic regression analyses were conducted. They were adjusted for confounding variables and after assessment of goodness-of-fit of the model, a scale parameter was added in order to account for over dispersion. Results are presented as absolute numbers, percentages, and odds ratios (OR). Data management and statistical analyses were performed using SAS EG 6.1 statistical software.

This study is part of a larger research project: “Prehospital management of stroke patients by the EMS”, which is protocolled online at www.clinicaltrials.gov (NCT02191514). Approval from Danish data protection agency (2007-58-0015) was obtained. Approval from the regional ethics committee was not needed (H-4-2014-FSP).

## Results

### Study population

We included 10,966 patients in the study: 4375 TIA’s and 6591 strokes. Of 2839 patients assessed for eligibility, 2653 were eligible for analysis. The dataflow is presented in Fig. 1.

Baseline characteristics for included patients are reported in Table 1. For characteristics of excluded data please see additional material [see Additional file 1].

**Table 1:** Characteristics for patients eligible for analysis

	Patients eligible for analysis (*n* = 2,653)	Recognised (*n* = 1,756)	Unrecognised (*n* = 897)
2012, *n* (%)	1,329 (50.1)	881 (50.2)	448 (49.9)
Male, *n* (%)	1,381 (52.1)	939 (53.5)	442 (49.3)
Age, median (IQR)	74 y (64–83)	74 y (64–83)	75 y (65–84)
Diurnal variation, *n* (%)			
-Day (07–15) -Evening (15–23) -Night (23–07)	1,575 (59.4) 812 (30.6) 266 (10.0)	1,067 (60.8) 529 (30.1) 160 (9.1)	396 (44.2) 378 (42.1) 123 (13.7)
Final ICD-10 diagnosis, *n* (%)			
-DG459 -DI61 -DI63	692 (26.1) 313 (11.8) 1,648 (62.1)	511 (29.1) 158 (9.0) 1,087 (61.9)	181 (20.2) 155 (17.3) 561 (62.5)

*Abbreviations*: *IQR* inter quartile range, *ICD-10* International Classification of Diseases, Tenth Revision

### Main results

#### Stroke recognition

For recognition of stroke and TIA, we found an overall sensitivity of 66.2 % (95 % CI: 64.4 %–68.0 %) and a PPV of 30.2 % (95 % CI: 29.1 %–31.4 %), see Table 2. Among the recognised cases, 79.3 % were categorised as A.26.03 (”*Lowered consciousness-paralysis, suspected stroke: Suddenly asymmetry in the face, lowered force in arms/legs, and/or verbal difficulties*”) and 20.7 % as A.26.04 (“*Lowered consciousness-paralysis, increasingly unclear/ listless-suspected stroke*”).

**Table 2:** Recognition of stroke by emergency medical dispatchers

		Final diagnosis of stroke/TIA		Total *N*, % (95 % CI)
		Yes	No	
Dispatch code for stroke/TIA	Yes	1,756	4,049	5,805
	No	897	128,766	129.663
Total		2,653	132,815	135.468
Sensitivity				66.2 % (64.4 %–68.0 %)
Positive predictive value				30.2 % (29.1 %–31.4 %)

*Abbreviations*: *TIA* transient ischemic attack, *Stroke* acute ischemic stroke or intracerebral haemorrhage, *CI* Confidence Interval

#### Factors associated with recognition of acute stroke

Recognition varied among diagnoses (TIA: 73.8 %, AIS: 66.0 %, and ICH: 50.5 %; *P < 0.0001*). Variation in stroke recognition according to the time of day (Daytime (07–15): 67.8 %, Evening (15–23): 65.2 %, and Night - time (23–07): 60.2 %; *P = 0.0401*), and sex (Male: 68 %, Female: 64.3 %; *P = 0.0406*) was detected. The multivariable logistic regression analyses were adjusted for sex, age, time of day and final diagnosis. This confirmed the results, however the effect of male sex on recognition diminished, see Table 3.

**Table 3:** Results of multivariable logistic regression model identifying factors associated with recognition of stroke/TIA

Effects	Unadjusted OR (95 % CI)	Adjusted OR (95 % CI)^a^
TIA vs. ICH	2.77 (2.10–3.66)	2.70 (2.04–3.57)
TIA vs. AIS	1.45 (1.20–1.78)	1.43 (1.15–1.78)
AIS vs. ICH	1.90 (1.49–2.43)	1.89 (1.48–2.41)
Day vs. Night	1.39 (1.07–1.82)	1.38 (1.02–1.87)
Day vs. Evening	1.12 (0.94–1.34)	1.12 (0.94–1.34)
Night vs. Evening	0.81 (0.61–1.07)	0.81 (0.61–1.09)
Age, y	0.994 (0.988–1.001)	0.996 (0.990–1.003)
Male	1.18 (1.01–1.39)	1.17 (0.99–1.38)

*Abbreviations*: *OR* odds ratio, *CI* confidence interval, *TIA* transient ischemic attack, *AIS* acute ischemic stroke, *ICH* intracerebral haemorrhage

^a^Adjusted for sex, age, time of day and final diagnosis

### Other analyses

#### Unrecognised and false positive cases

Among the unrecognised cases, 46.7 % were assigned the dispatch code “unclear problem” and 12.9 % were assigned the dispatch code “unconscious (lifeless) adult”. Please see additional material for detailed information about assigned dispatch codes for unrecognised cases [see Additional file 2].

Patients with suspected stroke by the EMD, but no record of stroke at hospital discharge, presented with a great variation of ICD-10 discharge diagnoses. The two most common were “Syncope and collapse” (3.2 %) and “Other and unspecified symptoms and signs involving the nervous and musculoskeletal systems” (3.0 %). Please see additional material for all diagnoses registered for false positive cases [see Additional file 2].

The unrecognised cases were dispatched as a priority A response in 53.1 % of cases and priority B in 46.6 %. Unrecognised cases with the “unclear problem” dispatch code assigned, were dispatched as a priority A response in 35.3 % of cases and priority B response in 64.5 % of cases, see Table 4.

**Table 4:** Response priority of unrecognised cases in total and for unrecognised cases dispatched as “unclear problem”

Priority	Unrecognised in total, *n* (%)	Unrecognised with “unclear problem” dispatch code, *n* (%)
A	476 (53.1)	148 (35.3)
B	418 (46.6)	270 (64.5)
C	3 (0.3)	1 (0.2)

Priority A: Acute, Priority B: Urgent, Priority C: Scheduled

## Discussion

### Key results

The study showed an overall sensitivity of 66.2 % and a PPV of 30.2 % for recognition of AIS, ICH, and TIA during emergency calls. The multivariable logistic regression analyses showed that a final diagnose of TIA and calls handled during the day were positively associated with recognition of acute stroke.

### Interpretation and generalisability

#### Stroke recognition

A sensitivity of 66.2 % is high compared to other reported results ranging from 31 % to 73.9 % [20–24]. However a PPV of 30.2 % is low compared other results ranging from 42 % to 59.1 % [21–23, 25]. The high sensitivity is likely the result of a profound reorganisation of the EMS Copenhagen during the past 5 years. This included, the introduction of EMDs with a medical profession (before it was police officers with no medical training) and the criteria-based dispatch tool Danish Index for Emergency Care. A recognition rate of 100 % is not obtainable without an inappropriate amount of false positive cases; however there is still room for improvement.

Early recognition of TIA is of outermost importance as the risk of developing a stroke after a TIA is substantial, especially in the first coming days [26]; and up to 23 % of AIS are preceded by a TIA [27]. Recognition of TIA provides an opportunity for early intervention and possible prevention of acute stroke.

#### Factors associated with recognition

The multivariable logistic regression analyses showed that calls during day-time, and final diagnoses of TIA and AIS were positively associated with recognition of acute stroke.

This finding is contrary to our expectations, as TIA was presumed more difficult to recognise as reported in another study [28]. This result is to our knowledge not reported elsewhere. The differences in recognition amongst final diagnoses, questions the otherwise general assumption that these conditions present similarly in patients. If any specific symptoms had a high predictive value for a specific pathology, it could facilitate the development of alternative prehospital scoring systems. Such could be of great value to the EMD, ambulance personnel, and the clinicians.

The diurnal variations in recognition of stroke could be explained by less awareness of stroke during the nights compared to day-time.

#### Unrecognised patients

Approximately 1/3 of the stroke/TIA patients were not recognised during the emergency call. In 47 % of the unrecognised cases, the EMD did not assign a priority A response, which may have delayed treatment for these patients, potentially impairing their outcome.

Among the unrecognised patients, the most present dispatch code was “unclear problem”. The unclear problem category is a necessity in the dispatch system due to the complexity of emergency calls. For the ongoing revaluation of the Danish Index for Emergency Care, it is essential to stress that 421 patients, with subsequently confirmed stroke or TIA, were assigned the “unclear problem” category during a 2-year period.

Among the unrecognised patients with a dispatch code of “unclear problem”, only 35.3 % received a priority A response, compared to 53 % among the unrecognised patients in total. This implies that when the EMD is in doubt, there is a tendency towards dispatching a less urgent response. This emphasises the importance of correct interpretation of symptoms.

#### False positive cases

With a PPV of 30.2 %, it is relevant to explore if certain conditions are often misinterpreted as a stroke by the EMD. The most common discharge diagnoses for false positive cases were “Syncope and collapse” and “Other and unspecified symptoms and signs involving the nervous and musculoskeletal systems”. However, these diagnoses only represented 3.2 and 3.0 % of false positive cases respectively, indicating that no specific conditions are systematically misinterpreted as stroke.

#### Future aspects

To decrease the prehospital delay, recognition by EMDs plays a major role and has the potential to reduce time from symptom onset to in-hospital evaluation; and thereby increase the proportion of patients receiving thrombolysis treatment.

Complete recognition is unobtainable, as the symptomatology is unspecific and differs among stroke patients. A higher recognition rate could though be obtained by encouraging the EMDs to suspect stroke in “not so obvious” cases. However this could result in over-dispatching, including an excessive number of dispatched priority A responses, resulting in under-prioritisation of other emergencies. Consequently, there would be an excessive risk of hazards and resource implications. In combination with the recognition rate, the PPV serves as an indicator of performance for international benchmarking between EMS organisations.

To achieve higher recognition rates without over-dispatching, different approaches can be considered. It might be speculated that EMDs with a medical background provides the best foundation for medical dispatch. It is eminent to strive for a high level of recognition through feedback and further education, focused on the complex task of medical dispatching. During EMD training courses, the prospects of fast and precise handling of potential stroke patients must be emphasised. In addition, internal audit of calls is a great learning tool and can be incorporated in the daily supervision program at the EMDC.

The use of Danish Index for Emergency Care as a decision tool for emergency calls is recently validated [14]. However, constant revision of this dispatch tool is necessary to ensure high performance. In such revisions, the introduction of a standardised stroke scale for evaluation of stroke symptoms would be highly relevant. Furthermore, the exploration of the “unclear problem” dispatch code could hold a great potential, as nearly half of the unrecognised patients in this study were assigned this dispatch code.

The importance of medical dispatch and recognition of acute conditions during emergency calls is acknowledged in the medical literature, especially considering out-of-hospital cardiac arrest [11, 29, 30]. National, as well as international, benchmarking on stroke recognition could serve as a quality indicator, and potentially contribute to better outcome for stroke patients.

### Limitations and strengths

Nearly 50 % of the stroke/TIA patients were unmatchable with EMDC data. It is reasonable to assume that these patients went to the hospital without calling the emergency number. This assumption is supported in the literature [31].

## Conclusion

This is an observational study of 2,653 emergency calls for patients suffering from AIS, ICH, or TIA, during a 2-year period from 2012 to 2014 in Copenhagen. The study reports an overall sensitivity of 66.2 % and a PPV of 30.2 % for recognition of AIS, ICH, and TIA during emergency calls. A final diagnose of TIA, compared to ICH, and calls handled during daytime were positively associated with recognition of acute stroke.

This study questions the otherwise general assumption that TIA, ICH, and AIS presents similarly in a clinical context, which could facilitate the development of supplementary prehospital scoring systems.

These results reports a potentially large group of stroke patients that are not recognised during the emergency calls, which could lead to prolonged time from symptom-onset to treatment and thereby worsening of outcome.

## Abbreviations

AIS, acute ischemic stroke; CI, confidence intervals; DSR, The Danish Stroke Registry; EMD, Emergency Medical Dispatcher; EMDC, Emergency Medical Dispatch Centre; EMS, Emergency Medical Services; ICD-10, International Classification of Diseases, Tenth Revision; ICH, intracerebral haemorrhage; IQR, inter quartile range; NPR, The National Patient Registry; OR, odds ratio; PPV, positive predictive value; SAH, subarachnoid haemorrhage; TIA, transient ischemic attack.

## Additional files

Additional file 1: Characteristics of total patient population and excluded patients within each step of exclusion (DOCX 19 kb) Additional file 2: Dispatch codes for unrecognised cases and ICD-10 discharge diagnoses for false positive cases (DOCX 15 kb)
