# Supplementary material for: Medical dispatchers recognise substantial amount of acute stroke during emergency calls
Source: Scand J Trauma Resusc Emerg Med. 2016 Jul 7;24:89. doi: 10.1186/s13049-016-0277-5 (PMC4936322; doi:10.1186/s13049-016-0277-5)
Supplement: Additional file 1: — Characteristics of total patient population and excluded patients within each step of exclusion (DOCX 19 kb) [file 13049_2016_277_MOESM1_ESM.docx]

|  | **NPR**  **(n=4,375)** | **DSR**  **(n=6,591)** | **Stroke and TIA in the study period* (n=10,909)** | **Unmatchable with EMDC data (n=5,043)** | **Ambulance acquired by other (n=3,027)** | **Missing dispatch code (n=186)** |
| --- | --- | --- | --- | --- | --- | --- |
| **2012, n (%)** | 2,182 (49.9%) | 3,269 (49.6%) | 5,419 (49.7%) | 2,469 (49.0%) | 1,528 (50.5%) | 93 (50.0%) |
| **Male, n (%)** | 2,274 (52.0%) | 3,399 (51.6%) | 5,636 (51.7%) | 2,633 (52.2%) | 1,523 (50.3%) | 99 (53.2%) |
| **Age, median (IQR)** | 69 y (58-78) | 73 y (64-82) | 71 y (61-81) | 68 y (59-78) | 73 y (64-82) | 70 y (56-80) |
| **Diurnal variation, n (%)** |  |  |  |  |  |  |
| - **Day (07-15)** - **Evening (15-23)** - **Night (23-07)** |  |  |  |  | 1,286 (42.5%)  899 (29.7%)  842 (27.8%) | 77 (41.4%)  80 (43.0%)  29 (15.6%) |
| **Final ICD-10 discharge diagnosis, n (%)** |  |  |  |  |  |  |
| - **DG459** - **DI61** - **DI63** | 4,375 (100%) | 722 (11.0%)  5,869 (89.1%) | 4,355 (39.9%)  720 (6.6%)  5,834 (53.5%) | 2,725 (54.0%)  201 (4.0%)  2,117 (42.0%) | 889 (29.4%)  182 (6.0%)  1,956 (64.6%) | 49 (26.3%)  24 (12.9%)  113 (60.8%) |

Additional file 1: Characteristics of total patient population and excluded patients within each step of exclusion.

Abbreviations: NPR: National Patient Registry, DSR: Danish Stroke Registry, Stroke: Acute Ischemic Stroke or Intracerebral Hemorrhage, TIA: Transient Ischemic Attack, Study period: 1^st^ of January 2012 to 31^st^ of December 2013, EMDC: Emergency Medical Dispatch Center, IQR: Interquartile Range, ICD-10: International Classification of Diseases, Tenth Revision, DG459: Transient Ischemic Attack, DI61: Intracerebral Hemorrhage, DI63: Acute Ischemic Stroke.
