# Supplementary material for: Medical dispatchers recognise substantial amount of acute stroke during emergency calls
Source: Scand J Trauma Resusc Emerg Med. 2016 Jul 7;24:89. doi: 10.1186/s13049-016-0277-5 (PMC4936322; doi:10.1186/s13049-016-0277-5)
Supplement: Additional file 2: — Dispatch codes for unrecognised cases and ICD-10 discharge diagnoses for false positive cases (DOCX 15 kb) [file 13049_2016_277_MOESM2_ESM.docx]

| Dispatch codes for unrecognized cases | | ICD-10 discharge diagnoses for false positive cases | |
| --- | --- | --- | --- |
| Dispatch codes | Unrecognized, n (%) | ICD-10 discharge diagnosis | False positive,  n (%) |
| Unclear problem (06) | 419 (46.7) | Syncope and collapse (DR559) | 108 (3.2) |
| Unconscious adult (01) | 116 (12.9) | Other and unspecified symptoms and signs involving the nervous and musculoskeletal systems (DR298) | 101 (3.0) |
| Chest pain – cardiac illness (10) | 99 (11.0) | Observation for suspected disease or condition, unspecified (DZ039) | 97 (2.9) |
| Lowered consciousness – paralysis (26) | 65 (7.3) | Symptoms from the nervous system, unspecified (DR298A) | 89 (2.7) |
| Accidents (33) | 39 (4.4) | Sequelae of stroke, not specified as haemorrhage or infarction (DI694) | 85 (2.5) |
| Wounds – fractures – minor injuries (31) | 39 (4.4) | Observation for other suspected diseases and conditions (DZ038) | 84 (2.5) |
| Breathing difficulties (28) | 36 (4.0) | Epilepsy, unspecified (DG409) | 77 (2.3) |
| Headache (19) | 25 (2.8) | Stroke, unspecified (DI649) | 71 (2.1) |
| Seizures (23) | 20 (2.2) | Other | 2,645 (78.8) |
| Other | 39 (4.4) | Missing | 692 |

Additional file 2: Dispatch codes for unrecognized cases and ICD-10 discharge diagnoses for false positive cases

Abbreviations: TIA: Transient Ischemic Attack, ICD-10: International Classification of Diseases, Tenth Revision.
